# Update on the Therapeutic Management of Hepatic Encephalopathy

**DOI:** 10.1007/s11894-018-0627-8

**Published:** 2018-04-11

**Authors:** Linda Skibsted Kornerup, Lise Lotte Gluud, Hendrik Vilstrup, Gitte Dam

**Affiliations:** 1 Department of Hepatology and Gastroenterology, Aarhus University Hospital, 44 Norrebrogade, 8000 Aarhus, Denmark; 2 Gastrounit, Medical Division, Copenhagen University Hospital, Kettegaard Allé 30, Hvidovre, 2650 Denmark

**Keywords:** Hepatic encephalopathy, Treatment, Lactulose, Rifaximin, Branched-chain amino acids, Probiotics

## Abstract

**Purpose of Review:**

Hepatic encephalopathy (HE) is a common and devastating complication to chronic liver disease. In this paper, we summarize the latest research and evidence of both conventional and up-coming treatments.

**Recent Findings:**

Meta-analyses report beneficial effects of lactulose, branched-chain amino acids, rifaximin, and to some degree l-ornithine l-aspartate on the manifestations of HE in patients with cirrhosis, and generally the numbers needed to treat are low. Recent studies on newer HE treatments including ornithine phenylacetate, spherical carbon, and fecal microbiota transplant also report potentially beneficial effects on HE manifestations.

**Summary:**

The conventional treatments benefit patients with HE. Newer treatments are under study and more research is needed for their validation.

## Introduction

Hepatic encephalopathy (HE) is a common complication of advanced liver disease. HE covers a complex set of non-specific neuropsychiatric symptoms and clinical signs affecting quality of life of both patients and their relatives. Due to frequent hospital contacts and admissions, HE is a major challenge to the healthcare system.

The pathophysiology of HE is not fully understood, but the condition reflects a diffuse disturbance of brain functions due to advanced liver disease or large portosystemic shunts (PSSs). The 2014 AASLD and EASL clinical practice guidelines for managing HE recommend classifying HE according to the underlying liver disease, the severity of the manifestations, the time course, and precipitating factors. HE may be divided into type A resulting from acute liver failure, type B resulting predominantly from PSS, and type C resulting from cirrhosis [1••]. Type C is by far the most predominant. HE describes a wide range of manifestations from minimal HE (MHE) over overt HE (OHE) to frank coma. OHE is present in 10–14% of patients at the time of diagnosis of cirrhosis. The 5-year risk of the first bout of OHE may be as high as 25%. Up to 40% of patients with cirrhosis will experience at least one episode; many will experience repeated episodes. MHE is diagnosed in 20–80% of patients with cirrhosis [2–6]. The 1-year incidence of type B OHE in patients with a transjugular intrahepatic portosystemic shunt (TIPS) is estimated to be 10–50% [7, 8]. HE is a very poor prognostic factor with a 1-year mortality of 54–85% [4, 9].

The initial treatment of patients with HE is directed at precipitating factors, present in at least 50%, and at general correction, stabilization, and nutrition. Several specific medical treatments against HE are available; most involve lowering of blood ammonia levels. This review provides an updated overview of the conventional and new drugs and principles for the treatment of HE (Table 1).

**Table 1:** Standard treatment and prevention schedule for HE

Treatment of an episode of OHE
Initial treatment
- Stabilization/correction
- Identification of precipitating factors
- Treatment of precipitating factors
- Nutrition
Specific treatment
- Lactulose
Treatment of MHE
- Nutrition
- Lactulose
- By failure: add-on rifaximin and/or BCAA
Prevention of recurrence of OHE or MHE
- Nutrition
- Lactulose
- By failure: add-on rifaximin and/or BCAA

## Old “Drugs,” New Evidence

### Lactulose

The non-absorbable disaccharide lactulose (and lactitol) is the first-line treatment for HE [10]. Acting as both osmotic laxative, prebiotic, and gut acidifying agent, lactulose causes several beneficial effects by both reducing the production and the absorption of ammonia from the intestines by changing the gut microbiota [11]. A 2016 updated Cochrane review found 31 randomized controlled trials investigating the treatment of HE, while 7 looked at HE prevention. Compared to placebo/no intervention, lactulose had a beneficial effect on HE with numbers needed to treat (NNT) of only four patients reflecting a marked relative risk reduction to 0.63. Lactulose had beneficial effects on both OHE and MHE, as well episodic as recurrent HE prevention, and also on the risk of serious adverse events such as liver failure, variceal bleeding, spontaneous bacterial peritonitis, hepatorenal syndrome, and ultimately on mortality [12••]. Lactulose is associated with only non-serious and mostly transient adverse events. Finally, lactulose is available all places and is very cheap. Thus, lactulose is now further established as the mainstay of prevention and treatment of all manifestations of HE.

### Branched-Chain Amino Acids

In cirrhosis, plasma levels of branched-chain amino acids (BCAAs: leucine, isoleucine, valine) are decreased as part of a general amino acid dyshomeostasis. There is increasing evidence for BCAAs being beneficial in HE, but the mechanism seems to be different from the originally assumed excitatory effect on neurotransmission. The effect is rather ammonia detoxification outside the liver via effects on skeletal muscle protein synthesis. Ammonia decreases protein synthesis by impairing the mTOR signaling, an effect counteracted by BCAAs [13].

A 2016 Cochrane review on the effects of BCAAs on HE in cirrhosis [14•] included 16 RCTs comprising 827 participants with HE classed as OHE (12 trials) or MHE (four trials). Eight trials assessed oral BCAA supplements, and seven trials assessed intravenous BCAAs. The control groups received placebo/no intervention (two trials), diets (ten trials), lactulose (two trials), or neomycin (two trials).

The meta-analyses showed that BCAAs have a beneficial effect on HE manifestations with a NNT of five patients and a relative risk reduction to 0.73. BCAAs had no effect on mortality.

The evidence shows that oral but not intravenous BCAAs have the beneficial effects. In sarcopenic patients with cirrhosis, the muscle build-up resulting from BCAAs, besides contributing to the effect on HE, carries important improvements in daily living and quality of life.

### l-Ornithine l-Aspartate

l-Ornithine l-Aspartate (LOLA) is the stable salt of the amino acids ornithine and aspartate. By at the same time providing metabolic substrates for urea cycle in liver and glutamine synthesis in skeletal muscle, LOLA stimulates ammonia detoxification and lowers blood ammonia [15]. A 2017 RCT compared the effect of intravenous LOLA versus placebo in reverting OHE at day 5 of treatment in a total of 193 patients with cirrhosis. The authors conclude that LOLA shortens the recovery time from OHE and the duration of hospitalization [16]. A meta-analysis of LOLA versus placebo or other interventions (lactulose, probiotics, and/or rifaximin) included 26 RCTs involving 1783 patients [17]. LOLA had a beneficial effect on HE (RR 0.60, 95% CI 0.44–0.82) and was associated with reduced mortality (RR 0.42, 95% CI 0.22–0.84). However, the data were highly selected and available for only 65% of eligible participants. Thus, the evidence for a beneficial effect of LOLA is weak. A definitive Cochrane review is underway.

### Non-absorbable Antibiotics

Antibiotics with activity against urease-producing gut bacteria have an ammonia-lowering effect. These include neomycin, paromomycin, metronidazole, vancomycin, and rifaximin. Rifaximin today is by far most commonly used owing to its low systemic absorption, broad antimicrobial spectrum, and low frequency of side effects [18]. The best evidence for the utility of rifaximin is the large 2010 RCT published in NEJM, where the drug as an add-on to patients with HE break-through despite correct lactulose treatment effectively protected against OHE recurrence [19•]. A 2014 review and meta-analysis of rifaximin versus placebo or other interventions (lactulose or other antibiotics) included 19 RCTs with a total of 1370 patients. Rifaximin had a beneficial effect on recovery from HE (RR 0.59; 95% CI 0.46–0.76), on secondary prevention of HE (RR 1.32; 95% CI 1.06–1.65), and on mortality (RR 0.68, 95% CI 0.48–0.97). The NNT was four patients [20]. Some of the included studies are small, but the results seem to be robust to bias control. Thus, rifaximin has a place mostly in prevention of recurrence of HE when lactulose alone fails. However, the accessibility of rifaximin may be limited by its high cost. Both neomycin and metronidazole have been used alone or as add-on to lactulose to treat HE. Generally, the studies are old and of low quality [19•, 21, 22]. Additionally, these antibiotics have serious side effects; neomycin causes nephrotoxicity, ototoxicity, and malabsorption; metronidazole causes irreversible peripheral neurotoxicity. Prolonged use is thus not recommended.

### Albumin

In advanced liver disease, the albumin production is reduced. Additionally, the albumin molecule may have decreased binding capacity and detoxification capacity [23–25]. The mechanism of action of albumin infusion in HE may be a combination of decreased oxidative stress and improved circulatory function. Few trials studied the effect of albumin on HE. A narrative review from 2015 suggested that albumin may have beneficial effects on HE recovery and mortality [26]. Albumin dialysis may also play an important role in improving HE in patients not responding to the best standard of care. In addition, an open-label RCT found beneficial effects of albumin as an add-on to lactulose on HE manifestations, the length of hospitalization, and mortality [27]. However, the trial was small and the results should be verified before any clinical conclusions can be made.

### Flumazenil

The role of flumazenil in the therapeutic management of HE is thought to be a reduction in the activity of the neuroinhibitory GABA/benzodiazepine receptor complex, countering the overall neural inhibition in HE [28]. A 2017 Cochrane meta-analysis concludes that flumazenil seems to have some short-term (minutes) beneficial effect on HE, but there is no evidence of effect on recovery, overall mortality, or health-related quality of life [29]. Flumazenil has no role in HE diagnostics.

### Hyperalimentation

The resting metabolic rate relative to lean body mass is often increased in patients with cirrhosis, which may contribute to their frequent malnutrition and sarcopenia [30, 31]. The patients exhibit reduced hepatic glycogen storage resulting in a faster switch to (obligatory) gluconeogenesis from amino acids and lipolysis when after a short period of fasting [32]. This consumption of blood amino acids promotes proteolysis, skeletal muscle wasting, increased dietary protein requirements, and increased ammonia production [33]. The 2013 International Society for Hepatic Encephalopathy and Nitrogen Metabolism guidelines for nutrition of HE patients [34] recommend a daily energy intake of 35–40 kcal/kg body weight including 1.2–1.5 g protein/kg body weight (best related to ascites-free body weight) for the maintenance and improvement of nutritional status. HE patients should eat small frequent meals evenly distributed during the day including a late night snack and avoid fasting for longer than 3–6 h during daytime. As mentioned above, coverage of some of the patients’ high-protein needs with BCAAs can have specific beneficial effects on sarcopenia and HE. Former times’ recommendation of low-protein diet to HE patients is definitively shown to be obsolete and harmful in increasing the risk for HE as well as for catabolism and risk for cirrhosis complications.

## New “Drugs,” New Evidence

### Ammonia-Lowering Agents

Ornithine phenylacetate (OPA), phenylbutyrate (PB), and benzoate act as ammonia scavengers by binding ammonia by their metabolism, leading to elimination of nitrogen by urinary non-urea excretion [35, 36].

*OPA* stimulates glutamine synthetase and thereby the formation of ammonia to glutamine. Glutamine subsequently combines with phenylacetate into phenylacetylglutamine (PAGN), eliminated in the urine [37]. A phase 2a study including 47 patients with acute liver injury or failure found OPA to be safe and well tolerated [38]. In agreement with previous findings, the study also suggests OPA to have a potential for dose-dependent ammonia lowering [39, 40]. There is at present no clinical evidence of a significant HE-ameliorating effect.

*PB*, a pro-drug of phenylacetate, also increases ammonia excretion in urine in the form of PAGN. A phase II RCT suggests that PB may lower ammonia and reduce the risk of new episodes of HE and hospitalizations in patients with earlier HE [41]. A preliminary study on PB to HE patients on intensive care unit indicated that PB might lower ammonia and improve HE status [42]. Presently, there is no definitive evidence of a significant HE-ameliorating effect.

*Benzoate* eliminates ammonia by conjugation with glycine to form hippurate, eliminated in urine [43, 44]. The drug is cheap and has few side effects. It is a time-honored remedy of congenital urea cycle defects. One small CRT found a comparable effect of sodium benzoate and lactulose in treating HE in patients with cirrhosis [44]. The drug is not established in the clinical management of HE but deserves further trials.

### Spherical Carbon (AST-120)

This is an orally administered, engineered carbon microsphere that adsorbs ammonia and other organic compounds from the gastrointestinal tract. In bile duct ligated rats, it lowers ammonia and decreases oxidative stress and brain edema [45]. Preliminary human studies report potential ammonia-lowering and cognitive benefits [46, 47].

### GABA_A_ Receptor Modulating Steroid Antagonists

A study on patients with cirrhosis and HE who died [48] found increased brain contents of neurosteroids. These increase activity in the strongly neuroinhibitory GABA system. Likewise, the brain of rats with chronic HE shows an increased GABAergic tone [49].

GABAA receptor modulating steroid antagonists (GAMSAs) act as antagonists to the positive GABA_A_ receptor, decreasing the effects of the neurosteroids [48]. This benefits arousal, cognition, and consciousness of patients with HE [50]. GAMSAs are shown to have positive effects on learning impairment in rats with experimental HE [51]. They also restore motor coordination, spatial learning, and memory in the rats [52]. The drug has successfully passed human safety trials and is now under clinical evaluation in cirrhosis patients with HE [50].

### Fecal Microbiota Transplant

Patients with cirrhosis tend to have a reduced abundance of several potentially beneficial bacterial families, including *Lachnospiraceae* and *Ruminococcaceae*, and an increased abundance of the pathogenic *Enterobacteriaceae* and *Streptococcaceae* [53, 54]. An open-label, randomized pilot trial on patients with recurrent HE compared the safety of fecal microbiota transplant (FMT) (from one donor matched to the dysbiosis in HE) versus no such intervention [55]. As a secondary trial outcome, the FMT was associated with fewer liver-related hospitalizations and improved cognitive function.

### Probiotics

Probiotics contain live microorganisms with expected beneficial effects on the gut dysbiosis and increased ammonia production in patients with cirrhosis and HE [56]. A Cochrane review concluded that probiotics may lower ammonia and improve recovery from HE and better the quality of life, but there was no clear improvement of overall mortality. However, most trials were of low quality with a high risk of systematic and random errors [57]. A comparison of the treatment effect of probiotics and the prebiotic lactulose was hence not possible.

## Other Treatments

Large portosystemic shunts (PSSs) may cause recurrent or persistent HE in patients with cirrhosis refractory to medical treatment. Embolization of the PSSs has been performed to improve HE in carefully selected patients, and a few retrospective studies are published within the last few years. A multicenter survey on 37 such patients showed more than half of the patients to be free of HE within 100 days of embolization and nearly half remained so for 2 years [58]. Similarly, a retrospective report [59] on 20 similar patients found that at all patients self-described sustained HE improvement; many of them could reduce or discontinue their anti-HE treatment, but about half had to continue their medical treatment. Thus, PSS embolization may be a treatment option in selected cases of persistent HE and complications to the ensuing portal hypertension such as esophageal varices allowing.

Liver transplantation represents the ultimate and in a way the only causal treatment of HE in patients with liver failure. Following transplantation, most patients are free of HE, but careful follow-up studies emphasize that some cognitive impairment may persist [60–63]. HE in itself is not considered an indication for transplantation.

## Conclusions

Until recently, the treatment of HE was rooted in strategies based upon personal experience, drug availability, and tradition in the respective hospital settings. As more RCTs are being performed and reported, more solid evidence is building up in support of marked beneficial effects of the conventional HE treatment strategies (lactulose, branched-chain amino acids, rifaximin) and confirms their central role in the specific HE treatment. Embolization of large PSSs and liver transplantation are efficient treatments in few and highly selected patients.

Additionally, newer and promising treatments are currently studied in experimental and clinical settings, but more evidence is needed before these treatments will earn a place in the routine HE treatment strategy. However, no revolutionary or causal treatment strategies have been introduced, probably due to the fact that the HE pathogenesis is still not fully understood.
